# Gremlin 1 depletion *in vivo* causes severe enteropathy and bone marrow failure

**DOI:** 10.1002/path.5450

**Published:** 2020-05-28

**Authors:** Simon C Rowan, Hanne Jahns, Liberty Mthunzi, Lucie Piouceau, Joanna Cornwell, Róisín Doody, Stephen Frohlich, John J Callanan, Paul McLoughlin

**Affiliations:** ^1^ University College Dublin, School of Medicine and Conway Institute Dublin Ireland; ^2^ University College Dublin, School of Veterinary Medicine Dublin Ireland; ^3^ Intensive Care Unit Beacon Hospital Dublin Ireland

## Abstract

The intestinal epithelium is perpetually renewed from a stem cell niche in the base of crypts to maintain a healthy bowel mucosa. Exit from this niche and maturation of epithelial cells requires tightly controlled gradients in BMP signalling, progressing from low BMP signalling at the crypt base to high signalling at the luminal surface. The BMP antagonist gremlin 1 (*Grem1*) is highly expressed by subepithelial myofibroblasts adjacent to the intestinal crypts but its role in regulating the stem cell niche and epithelial renewal *in vivo* has not been explored. To explore the effects of *Grem1* loss in adulthood following normal growth and development, we bred mice (*ROSA26CreER‐Grem1* ^*flx/flx*^) in which *Grem1* could be deleted by tamoxifen administration. While *Grem1* remained intact, these mice were healthy, grew normally, and reproduced successfully. Following *Grem1* depletion, the mice became unwell and were euthanised (at 7–13 days). Post‐mortem examination revealed extensive mucosal abnormalities throughout the small and large intestines with failure of epithelial cell replication and maturation, villous atrophy, and features of malabsorption. Bone marrow hypoplasia was also observed with associated early haematopoietic failure. These results demonstrate an essential homeostatic role for gremlin 1 in maintaining normal bowel epithelial function in adulthood, suggesting that abnormalities in gremlin 1 expression can contribute to enteropathies. We also identified a previously unsuspected requirement for gremlin 1 in normal haematopoiesis. © 2020 The Authors. *The Journal of Pathology* published by John Wiley & Sons Ltd on behalf of Pathological Society of Great Britain and Ireland.

## Introduction

The intestinal mucosa is covered by a perpetually self‐renewing layer of epithelium that is sustained by intestinal epithelial stem cells in each crypt’s base. This stem cell niche is tightly controlled by key regulatory signals that are involved in intestinal stem cell renewal and differentiation, including Wnt, bone morphogenetic protein (BMP), and notch pathways [1, 2]. Strict gradients of interacting morphogens control the exit of stem cell progeny from the niche driven by high Wnt and low BMP signalling in the lower half of the crypt, before progressively differentiating into post‐mitotic specialised cells, controlled by low Wnt and high BMP signalling at the luminal surface [3].

A number of proteins that antagonise BMP functions including gremlin 1 (GREM1), gremlin 2 (GREM2), and noggin (NOG) are expressed locally by subepithelial myofibroblasts adjacent to the intestinal crypts. *In vitro* BMP antagonists are essential for culture of intestinal organoids or crypts, suggesting that the normal Wnt–BMP gradient requires the restricted paracrine secretion of these antagonists [4, 5]. Of these BMP antagonists, gremlin 1, encoded by *GREM1*, which blocks BMP‐2,BMP‐4, and BMP‐7 signalling, has been most directly linked to disease pathogenesis [6]. Human mixed polyposis syndrome is caused by a 40 kbp genetic duplication that results in excessive epithelial expression of *GREM1* mRNA and transgenic overexpression of *Grem1* initiates intestinal tumourigenesis in animal models [4, 7]. Furthermore, *GREM1* mRNA is highly expressed in the stroma of common colon cancers [7, 8]. Taken together, those studies suggest that reducing *GREM1* mRNA (and thus protein) expression or inhibiting GREM1 function may be a novel therapeutic strategy in intestinal disorders characterised by dysregulated Wnt–BMP signalling or aberrant gremlin 1 expression.

In contrast to the well‐described role of increased *GREM1* mRNA in disease pathogenesis, no studies to date have reported pathogenetic effects of reduced *GREM1* mRNA in the bowel. The importance of the individual BMP antagonists in intestinal homeostasis *in vivo* has not previously been investigated. To assess the role of *Grem1* in intestinal epithelial homeostasis, we induced widespread *Grem1* deletion in adult mice, at a stage when normal growth and development were complete, by using tamoxifen‐activated cre recombinase (*ROSA26CreER‐Grem1* ^*fl/fl*^), thus circumventing the perinatal lethality caused by complete loss of *Grem1 in utero* [9]. Our results demonstrate that *Grem1* plays an indispensable role in maintaining the normal bowel epithelium. Unexpectedly, we also discovered an essential role for *Grem1* in haematopoiesis.

## Materials and methods

Adult male mice (sexually mature, 3–6 months old) expressing tamoxifen‐activated cre recombinase driven by the ubiquitously expressed *ROSA26* promoter and in which the coding sequence of both alleles of *Grem1* had been flanked by loxP sites (*ROSA26CreER‐Grem1* ^*fl/fl*^) were given dietary tamoxifen to induce *Grem1* depletion [10, 11]. At the end of the experimental protocols, mice were deeply anaesthetised and then euthanised by cervical dislocation. Systematic post‐mortem examinations were undertaken and tissues fixed for histological examination. Marrow was isolated from the femur and tibia to count total cell numbers. In further mice, organs were removed post‐mortem and immediately flash frozen for later analysis of mRNA. All protocols were approved by the UCD Animal Research Ethics Committee and licensed by the Department of Health, Ireland. Detailed methods may be found in supplementary material, and methods.

## Results

**Figure 1:**
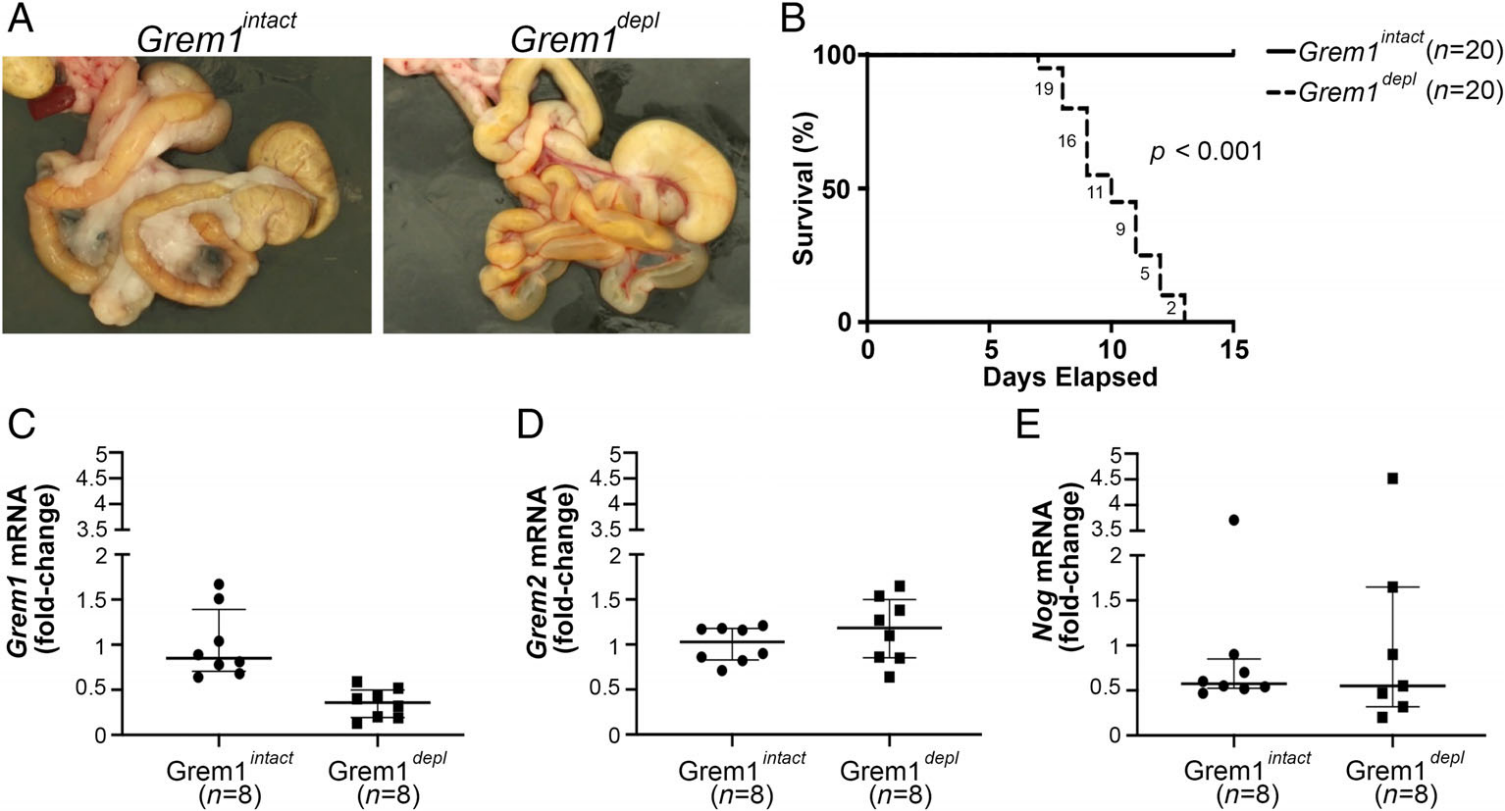
Ubiquitous *Grem1* deletion in adulthood is lethal and the effects manifest acutely in the bowel. (A) Representative image of the normal intestinal tract of a gremlin 1 (*Grem1*) intact (*Grem1* ^*intact*^) mouse and an image of an abnormal intestine from a *Grem1* depleted mouse (*Grem1* ^*depl*^) following exposure to tamoxifen. It shows thin transparent intestinal walls distended by clear pale yellow gelatinous material and a loss of mesenteric fat. (B) Kaplan–Meier survival curves of *ROSA26CreER‐Grem1* ^*fl/fl*^ mice on a normal diet (*Grem1* ^*intact*^, *n* = 20) or a tamoxifen diet (*Grem1* ^*depl*^, *n* = 20, *p* < 0.001, log‐rank test). Numbers on the graph indicate the number of mice remaining at the end of each day of the tamoxifen diet. (C) *Grem1* mRNA assayed by RT‐qPCR in small intestines of *Grem1* intact (*Grem1* ^*intact*^, *n* = 8) and *Grem1* depleted (*Grem1* ^*depl*^, *n* = 8) mice. (D) Gremlin 2 (*Grem2*) mRNA assayed by RT‐qPCR in small intestines of *Grem1* intact (*Grem1* ^*intact*^, *n* = 8) and *Grem1* depleted (*Grem1* ^*depl*^, *n* = 8) mice. (E) Noggin (*Nog*) mRNA assayed by RT‐qPCR in small intestines of *Grem1* intact (*Grem1* ^*intact*^, *n* = 8) and *Grem1* depleted (*Grem1* ^*depl*^, *n* = 8) mice.

In the absence of tamoxifen, adult *ROSA26CreER‐Grem1* ^*fl/fl*^ mice remained well for periods up to 18 months, successfully mated, and produced healthy offspring with equal numbers of male and female pups. When adult mice were fed tamoxifen to induce gremlin 1 depletion (*Grem1* ^*depl*^), they initially appeared healthy. However, after 12–13 days of administration the first four mice given tamoxifen died unexpectedly. Post‐mortem examination of these revealed markedly dilated intestines, with a translucent intestinal wall, that contained watery yellow or gelatinous fluid mixed with small amounts of white digesta (Figure 1A).

Following this, an intensive monitoring regimen was instituted, and any mice given tamoxifen that lost more than 25% of body weight or appeared unwell were euthanised after periods ranging from 7 to 12 days. All of the matched *ROSA26Cre‐Grem1* ^*fl/fl*^ mice in the control groups fed a normal diet (*Grem1* ^*intact*^) remained well (Figure 1B). *Grem1* mRNA expression was markedly reduced (Figure 1C) in the intestines of *Grem1* ^*depl*^ mice (*n* = 8) when compared with the *Grem1* ^*intact*^ mice (*n* = 8). Expression of *Grem2* and Noggin (*Nog*), the two other BMP antagonists that are expressed in intestinal crypts and also block BMP‐2,BMP‐4, and BMP‐7, was unchanged [12, 13]. Wild‐type mice and mice expressing the tamoxifen‐inducible ROSA26 Cre recombinase alone (i.e. in the absence of floxed *Grem1* alleles) remained well during tamoxifen administration (data not shown), in keeping with previous reports [14].

**Figure 2:**
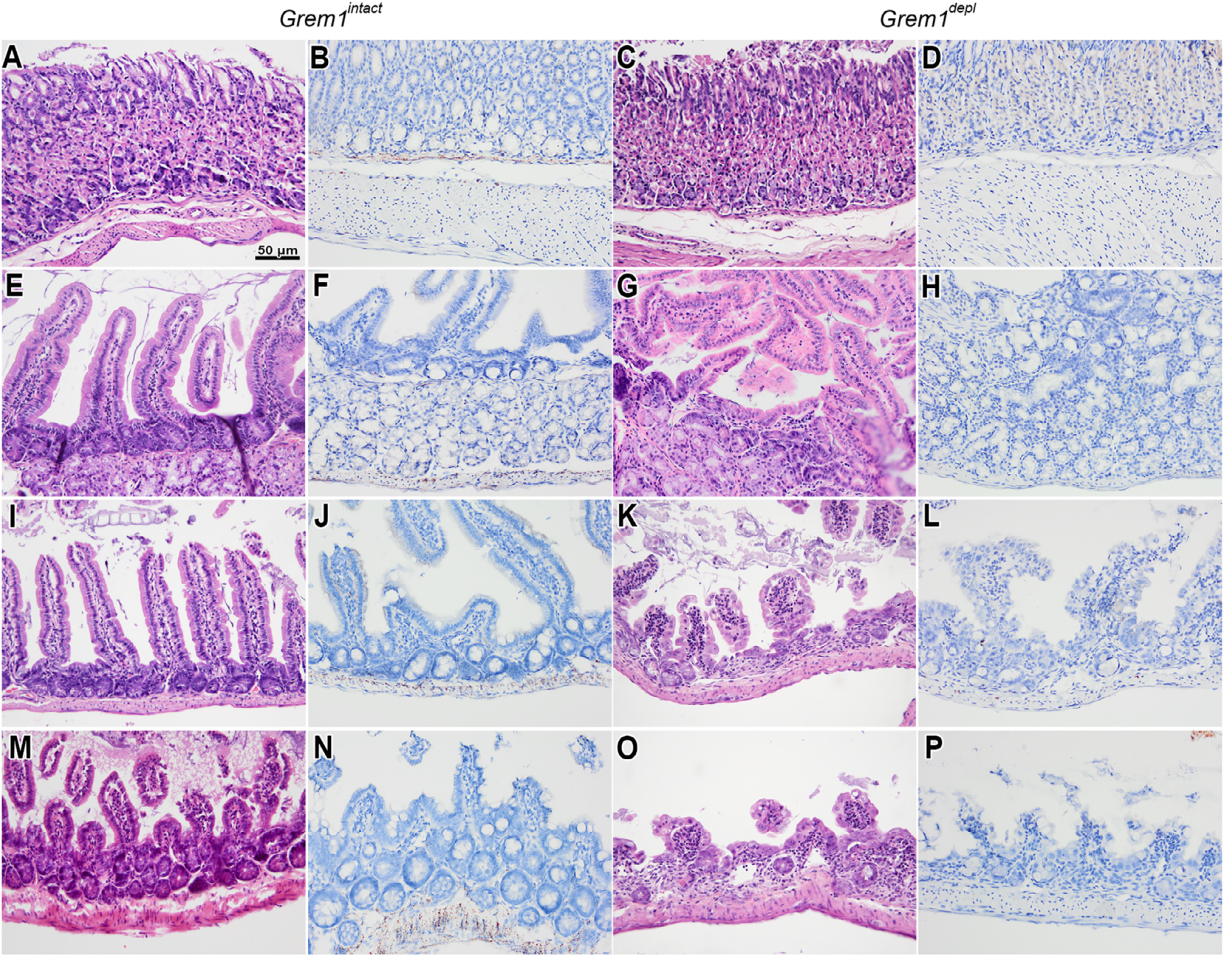
Attenuated *Grem1* expression was associated with epithelial cell abnormalities, villus stunting, and loss of crypts in the small intestines of *Grem1* ^*depl*^ mice. (A–P) Representative images of the stomach (first row) and small intestine (rows 2–4) of gremlin 1 intact (*Grem1* ^*intact*^ *)* (first and second columns) and gremlin 1 depleted (*Grem1* ^*depl*^) mice (third and fourth columns). (A–D) The stomachs of both *Grem1* ^*intact*^ and *Grem1* ^*depl*^ mice were normal. (E–P) The intestine of *Grem1* ^*depl*^ mice showed marked villus stunting and multifocal collapse and loss of crypts in the duodenum (E–H), jejunum (I–L), and ileum (M–P) in comparison to *Grem1* ^*intact*^ controls. The mucosal epithelial lining of the intestine consisted of disordered, large polygonal cells. (B, F, J, N) *In situ* hybridisation (ISH) revealed *Grem1* mRNA expression as punctate brown labelling outside the base of the crypts and along the muscularis mucosa of the stomach (B) and intestine (F, J, N) of *Grem1* ^*intact*^ mice. Similar but less intense labelling was shown in the submucosa and the tunica muscularis. (D, H, L, P) In contrast, no *Grem1* mRNA labelling was found in the stomach (D) or intestine (H, L, P) of *Grem1* ^*depl*^ mice. First and third columns, H&E staining; second and fourth columns, ISH for *Grem1* mRNA, counterstained with Mayer’s haematoxylin. Scale bar = 50 μm (20× objective, numerical aperture 0.75).

A group (*n* = 8) of *Grem1* ^*depl*^ mice was examined post‐mortem and compared with *Grem1* ^*intact*^ mice (*n* = 6). Dilated translucent intestines filled with clear or yellow gelatinous material were observed in four of the eight *Grem1* ^*depl*^ mice, similar to the four mice initially exposed to tamoxifen that had died unexpectedly. Histopathological examination of the stomach was normal (Figure 2). However, examination of the duodenum, jejunum, and ileum revealed marked villous stunting and loss (Figure 2). The epithelial lining was mainly intact but consisted predominantly of large polygonal and cuboidal cells of varying sizes with abundant cytoplasm and large, centrally located nuclei and evidence of arrested cell proliferation (supplementary material, Figure S1). Crypts were collapsed or lost at multiple sites, while those that remained were lined by abnormal epithelial cells and frequently had no lumen (Figure 2). Mitotic figures were rarely observed in the crypt‐lining cells of *Grem1* ^*depl*^ mice and there was marked reduction of Ki67 expression, indicating reduced cell replication (supplementary material, Figure S1C,D).

**Figure 3:**
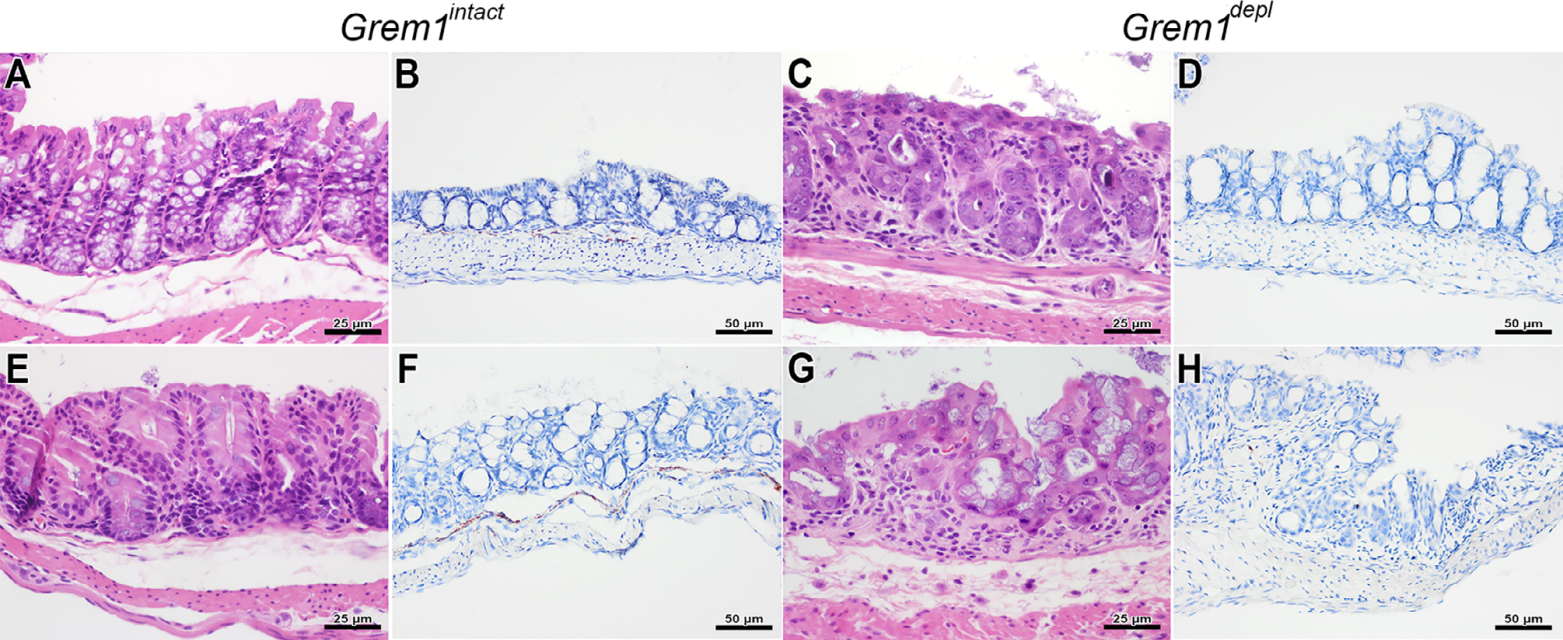
*Grem1* depletion induced widespread changes in the large intestinal epithelium, with superficial, abnormal crypts that were reduced in number. (A–H) Representative images of the caecum (first row) and colon (second row) of gremlin 1 intact (*Grem1* ^*intact*^) mice (first and second columns) and gremlin 1 depleted (*Grem1* ^*depl*^ *)* mice (third and fourth columns) illustrate the simple columnar epithelial cells and goblet cells in *Grem1* ^*intact*^ mice which were replaced by large polygonal cells with abundant eosinophilic cytoplasm and a centrally located large round‐to‐oval nucleus in *Grem1* ^*depl*^ mice. (A, C, E, G) Crypts were pushed to the surface or lost in the caecum and colon of *Grem1* ^*depl*^ mice in comparison to the caecum and colon of *Grem1* ^*intact*^ mice. (B, F) *In situ* hybridisation demonstrated *Grem1* mRNA expression as punctate brown labelling at the base of the crypts, along the muscularis mucosa, with occasional brown dots in the submucosa and the tunica muscularis of the caecum and colon of *Grem1* ^*intact*^ mice. (D, H) In contrast, no *Grem1* mRNA labelling was found in the caecum or colon of *Grem1* ^*depl*^ mice. First and third columns, H&E staining, scale bar = 25 μm (40× objective, numerical aperture 0.95); second and fourth columns, ISH for *Grem1* mRNA, counterstained with Mayer's haematoxylin, scale bar = 50 μm (20× objective, numerical aperture 0.75).

The caecum and colon of *Grem1* ^*depl*^ mice showed similar widespread changes of the epithelial cells, with varying mucosal thickness and more superficial, abnormal crypts (Figure 3 and supplementary material, Figure S1). Mitotic figures were infrequent in the crypt epithelium. Staining for proteoglycans (Alcian blue) in the large intestine illustrated a loss of regularly arranged goblet cells (supplementary material, Figure S1E–H). *Grem1* ^*intact*^ mice showed normal mucosal structures in the caecum and colon. Serum urea, creatinine, and cholesterol were significantly reduced in *Grem1* ^*depl*^ mice (supplementary material, Table S1), compatible with malabsorption and loss of muscle mass.

In *Grem1* ^*intact*^ mice, *Grem1* mRNA expression was seen in pericryptal fibroblasts and in the muscularis mucosa along the entire gastrointestinal tract but most prominently in the ileum (Figures 2 and 3). Similar, but less intense *Grem1* ISH staining was observed in stromal cells of the submucosa and in the tunica muscularis (supplementary material, Figure S2), in keeping with previous findings in the normal gastrointestinal tract [4, 5, 7, 8, 15, 16]. In the *Grem1* ^*depl*^ mice, *Grem1* expression was markedly and extensively reduced throughout the gastrointestinal tract (Figures 2 and 3), although *Grem1* expression was seen occasionally in small clusters of cells (supplementary material, Figure S3B,D).

**Figure 4:**
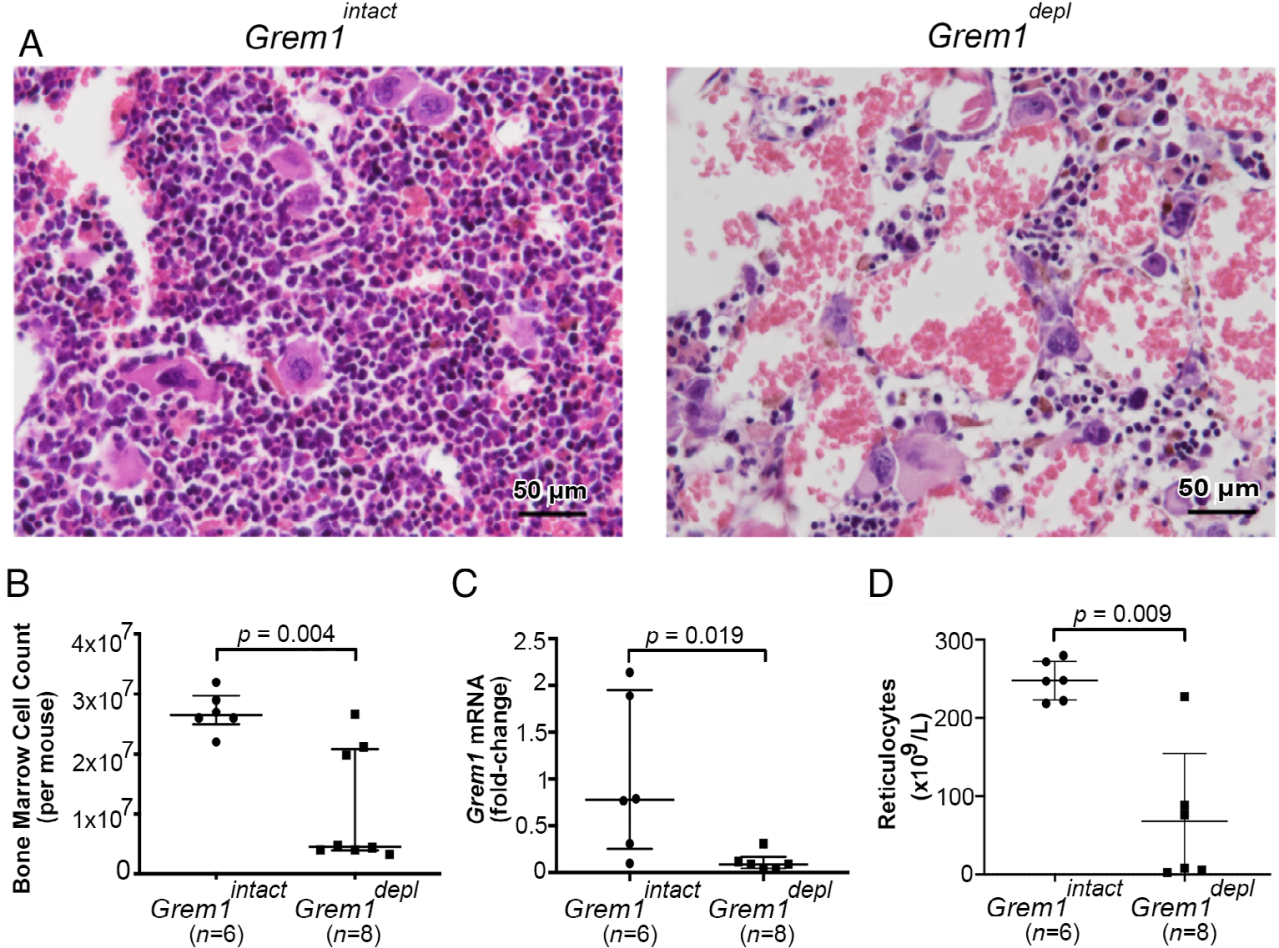
*Grem1* is essential for normal bone marrow function. (A) Image of the normal bone marrow of a *Grem1* intact (*Grem1* ^*intact*^) mouse (left) showing marked cellular reduction, with replacement of the marrow by large blood‐filled sinuses in a *Grem1* depleted (*Grem1* ^*depl*^) mouse (right). H&E, scale bar = 25 μm (40× objective, numerical aperture 0.95). (B) Total number of cells obtained from the long bones of the legs of *Grem1* ^*intact*^ (*n* = 6) and *Grem1* ^*depl*^ (*n* = 8) mice. (C) *Grem1* mRNA expression in bone marrow‐derived macrophages of *Grem1* ^*intact*^ (*n* = 6) and *Grem1* ^*depl*^ (*n* = 6) mice. (D) Peripheral blood reticulocyte count in *Grem1* ^*intact*^ (*n* = 6) and *Grem1* ^*depl*^ (*n* = 6) mice. Median (IQR) relative to mean value in the *Grem1* ^*intact*^ group. Statistical comparisons were made using a Mann–Whitney *U*‐test.

The second major abnormality noted in *Grem1* ^*depl*^ mice was that the four mice affected by the intestinal changes showed severe depletion of myeloid, lymphoid, and erythroid lineages in the bone marrow with replacement of cells by large vascular sinuses, an appearance similar to aplastic anaemia (Figure 4A). Only scattered small groups of progenitor cells and megakaryocytes remained in the bone marrow. The number of bone marrow cells isolated from *Grem1* ^*depl*^ mice was significantly less than that from *Grem1* ^*intact*^ mice (Figure 4B). Bone marrow cells obtained from the *Grem1* ^*depl*^ mice had reduced *Grem1* mRNA expression when compared with *Grem1* ^*intact*^ mice (Figure 4C). The peripheral blood reticulocyte count (Figure 4D) was reduced in *Grem1* ^*depl*^ mice compared with *Grem1* ^*intact*^ mice (supplementary material, Table S2), compatible with the early stages of failure of erythropoiesis.

Histological examination of the liver, kidneys, adrenal glands, spleen, testes, lungs, and heart was normal in all *Grem1* ^*depl*^ mice (data not shown).

## Discussion

Our findings show for the first time that gremlin 1 plays an essential role in the maintenance of mucosal function in the adult bowel *in vivo*. Taken together with previous evidence that BMP signalling is required for maintenance of normal bowel structure and function [17], our results demonstrate that carefully regulated interactions of both the ligands and the antagonist are needed for a healthy bowel *in vivo*. Disruption of this balance may contribute to tumour development, as previously demonstrated, but may also play a role in enteropathies characterised by mucosal atrophy and failure.

The severe disruption of bowel structure and function that we observed after *Grem1* depletion contrasts sharply with the absence of bowel disturbance reported by Davis *et al* [4] following *Grem1* depletion induced using a different, tamoxifen‐induciblecre‐recombinase(CAGG‐CreErT2), which was at least as effective as the ROSA26‐CreERT2 that we used. Differences in the genetic backgrounds of the different mouse colonies may explain the very different phenotypes. Another potential explanation for the differences is that the bowel abnormalities were secondary to immunosuppression caused by the bone marrow failure that we found. This failure was unexpected since even though *Grem1* is expressed in the normal marrow [15, 18], a requirement for *Grem1* in normal haematopoiesis has not previously been reported. However, the peripheral blood white cell counts were unchanged at the time our mice became unwell and histopathological examination did not show the epithelial ulceration, bacterial invasion, and necrosis typical of neutropenic enterocolitis [19]. Interestingly, in a paper published while our manuscript was under review, McCarthy *et al* [20] reported that ablation of *Grem1*‐expressing cells in the small intestinal wall (achieved using a tamoxifen‐inducible diphtheria toxin receptor regulated by the *Grem1* promoter) produced a small intestinal phenotype closely similar to that which we report. Taken together with our data, this suggests that the bowel abnormalities were a result of the direct effects of *Grem1* loss in the bowel. It is also worth noting that although Davis *et al* [4] did not find any bowel abnormality in the absence of other genetic mutations, they observed a reduced polyp burden following *Grem1* depletion in mice carrying a tumour‐promoting mutation of the adenomatous polyposis coli locus, a finding compatible with the role for *Grem1* in the regulation of epithelial proliferation that we found.

The abnormalities caused by *Grem1* depletion were seen only in tissues with very rapid turnover, i.e. bowel epithelium and bone marrow cells. Thus, even though we found no abnormalities in any other organs, *Grem1* may have important functions in those organs that were not manifested because of the rapidly fatal effects of *Grem1* loss in the bowel and marrow.

In summary, our results demonstrate for the first time an essential homeostatic role for gremlin 1 in the maintenance of normal bowel function in adulthood *in vivo* and demonstrate a previously unsuspected, essential requirement for normal gremlin 1 expression in bone marrow function. Moreover, our findings show that gremlin 1 is non‐redundant among the BMP antagonists. Taken together, our findings suggest that abnormally reduced or increased expression of gremlin 1 may play a significant role in disease development in the gastrointestinal tract and bone marrow.

## Author contributions statement

SCR and PMcL were responsible for conceptualisation. SCR, JC, LP, LM, and PMcL were responsible for methodology. SCR, HJ, LM, JC, LP, RD, SF, and JJC conducted investigations. SCR, HJ, LP, and PMcL wrote the original draft. SCR, HJ, LM, LP, JC, RD, SF, and JJC reviewed and edited the manuscript. SCR, HJ, JC, LP, and PMcL were responsible for visualisation. SCR and PMcL were in charge of project administration. PMcL acquired funding.

## Supporting information


**Supplementary materials and methods**

**Supplementary figure legends**

**Figure S1.** *Grem1* depletion induces widespread changes to the intestinal epithelium and reduces indices of proliferation. Representative images of the (A–D) jejunum, (E, F) caecum, and (G, H) colon of *Grem1* ^*intact*^ (first column) and *Grem1* ^*depl*^ mice (second column)

**Figure S2.** *Grem1* mRNA expression in stromal cells throughout the gastrointestinal tract in *Grem1* ^*intact*^ mice. Representative images of ISH for *Grem1* mRNA in the (A) stomach, (B) duodenum, (C) jejunum, (D) ileum, (E) caecum, and (F) colon of *Grem1* ^*intact*^ mice

**Figure S3.** *Grem1* mRNA expression was markedly and extensively reduced in the gastrointestinal tract of *Grem1* ^*depl*^ mice, although some staining was occasionally evident**.** Representative images of the intestine of *Grem1* ^*intact*^ (first column) and *Grem1* ^*depl*^ mice (second column)

**Table S1.** Results of the serum biochemical analyses
**Table S2.** Results of peripheral blood haematological analyses
