# Supplementary material for: Gremlin 1 depletion in vivo causes severe enteropathy and bone marrow failure
Source: J Pathol. 2020 May 28;251(2):117–22. doi: 10.1002/path.5450 (PMC7384058; doi:10.1002/path.5450)
Supplement: Supplementary file 1 — Supplementary materials and methods [file PATH-251-117-s001.docx]

**Gremlin 1 depletion *in vivo* causes severe enteropathy** **and bone marrow failure**SC Rowan, H Jahns, *et al. J Pathol* DOI: 10.1002/path.5450

**Supplementary materials and methods**

Reference numbers refer to the main text list

*Generation of* Grem1^fl/fl^ *mice*

*Grem1^flx/+^* mice (generously provided by Dr Aris Economides through Regeneron, New York, USA; VelociGene modified allele ID number 1083), as previously described [11], were mated with *B6;129-Gt(Rosa)26Sor^tm2(icre/ERT2Nat)^/J* mice (Stock number 004847; Jax Laboratories, Maine, USA), in which tamoxifen-inducible cre recombinase expression was driven by the ubiquitously expressed *ROSA26* promoter to generate mice (*ROSA26Cre-Grem1^fl/fl^*) in which *Grem1* deletion could be induced in all cells by tamoxifen administration [21]. Adult male and female mice homozygous for both the ROSA26Cre and the *Grem1^fl/fl^* alleles (here called *ROSA26Cre-Grem1^fl/fl^* mice) were used in all experiments and were housed under specific pathogen-free conditions. The *B6;129-Gt(Rosa)26Sor^tm2(icre/ERT2Nat)^/J* mouse strain used to develop these *ROSA26Cre-Grem1^fl/fl^* mice has previously been shown to induce effective recombination in the gastrointestinal tract [14,22]. All experimental protocols and procedures were approved by the UCD Animal Research Ethics Committee and licensed by the Department of Health, Ireland.

*Genotyping of mice*

Genotyping of mice during breeding was undertaken by PCR analysis of genomic ear DNA, using standard methods. The presence of the wild-type gremlin 1 allele was detected using the common reverse primer 5'-TTAAAACAGGAGTGGTCAGCA-3' together with the forward primer 5'-GACATCTCAGTCGTAGCCCA-3' to produce an amplicon of 441 bp. The floxed gremlin 1 allele was detected using the common reverse primer together with the forward primer 5'-GGTGGGGTGGGATTAGATA-3' to produce an amplicon of 670 bp.

Deletion of the LoxP flanked sequence was detected using the forward primer 5'-AGGTTTCCTGGGCTTTTTCCA-3' together with the reverse primer 5'-AGTGGTCAGCATTTCACCCT-3', which produced an amplicon of 348 bp following tamoxifen-induced deletion of the LoxP flanked segment.

To detect the presence of the native ROSA26 sequence, the common forward primer (5'-AAGGGAGCTGCAGTGGAGTA-3') was used in combination with reverse primer (5'-CCGAAAATCTGTGGGAAGTC-3') to detect a 145 bp amplicon, while the mutated ROSA26 cre recombinase allele was detected using the reverse primer 5'-TCTTGCGAACCTCATCACTC-3' allele to detect the presence of a 237 bp amplicon.

*Tamoxifen administration*

Excision of *Grem1* sequences in *ROSA26Cre-Grem1^fl/fl^* mice was induced by providing *ad libitum* access to commercially available tamoxifen-containing food pellets (400 mg/tamoxifen citrate/kg diet; Envigo, Huntingdon, UK). Controls were provided *ad libitum* access to a normal rodent diet for an identical duration. Animals were randomly allocated to receive either a normal (tamoxifen-free) diet or food pellets containing tamoxifen by using a random number generator to assign each mouse to a group. During this period, the wellbeing of the mice was monitored closely using a standard scoring system incorporating appearance, grooming, respiration, behaviour, stools, and body weight, and mice were euthanised if the combined score exceeded a specific value (humane endpoint) or when the mice had been exposed to the diet for a period planned in a specific experimental protocol, whichever occurred earlier (see the Results section). Euthanasia was by induction of anaesthesia with inhaled isoflurane (5% in oxygen) followed by cervical dislocation. Mice in the matched groups fed a normal diet were euthanised after a period planned for each specific protocol (see the Results section).

*Post-mortem examination and tissue collection*

In groups of mice selected for detailed post-mortem examination, a blood sample was collected immediately after euthanasia and anti-coagulated with EDTA (Sarstedt, Nümbrecht, Germany) at 4 °C for analysis of whole blood count (Advia 2120 Hematology System, Siemens Healthcare GmbH, Erlangen, Germany). A second sample was collected without anti-coagulant (serum tube; Sarstedt), allowed to clot at 4 °C, and the serum separated by centrifugation (2795 × *g* for 15 min) for biochemical analyses. The serum was transferred into a fresh Eppendorf and stored at −80 °C for later analysis. All stored serum samples were analysed on the same day using an automated analyser (Clinical Chemistry Analyser RX Imola, Randox, Crumlin, Ireland).

A systematic post-mortem examination was undertaken using a standard protocol [23]. In brief, a midline incision was made and the thorax was opened by removing the sternum. The adrenal glands, heart, intestines, kidney, liver, lungs, spleen, stomach, testes, and thymus were removed and weighed using a fine balance (Mettler Toledo, Columbus, OH, USA). All tissues were assessed macroscopically and placed in fixative (10% vol/vol buffered formalin) for 24 h before embedding in paraffin wax (automated tissue processor; Tissue Tek, Sakura Finetek, Torrance, CA, USA) and later sectioned (4–5 μm) for histological examination. In a separate group of mice, the same organs were removed post-mortem and immediately flash-frozen for later analysis of mRNA.

The hind limbs were removed following euthanasia by transection through the hip joint. The superficial tissue surrounding the bones was removed and one femur immediately fixed for histological examination. One femur and the unopened skull were decalcified using a Shandon™ TBD-1 Decalcifier (Thermo Fisher Scientific, Waltham, MA, USA) for 18 h. Five transverse sections of the head and femur were then embedded in wax for histological examination. To assess haematopoietic stem cell numbers, the remaining femur and the two tibias were briefly immersed in ethanol (70% vol/vol) for disinfection and then placed in ice-cold PBS. In a laminar flow hood, the epiphyses were removed, the marrow cavities flushed with PBS, and the cells collected. This cell suspension was passed through a cell strainer (70 µm) to remove debris and centrifuged (400 × *g* for 4 min at 4 °C); the supernatant was removed and the pellet resuspended in red blood cell lysis buffer (1 ml of solution containing ammonium chloride 155 mm, potassium bicarbonate 10 mm, and EDTA 0.1 mm) for 1 min. PBS (9 ml) was then added to the suspension to stop further lysis; the suspension was centrifuged (400 × *g* for 4 min at 4 °C), the supernatant removed, and the cells were resuspended in DMEM (Thermo Fisher Scientific) supplemented with L929-cell conditioned medium (20% vol/vol), fetal bovine serum (FBS, 10% vol/vol; Sigma-Aldrich, Dublin, Ireland), and HEPES (10 mm; Sigma-Aldrich). The cells isolated from the bone marrow were then counted using a haemocytometer. The bone marrow progenitor cells were then cultured in Dulbecco’s modified Eagle’s medium (DMEM; Sigma-Aldrich) supplemented with L929 (mouse fibroblast cell line)-conditioned medium (20% vol/vol) and FBS (10% vol/vol) for 7 days. Cells were then homogenised in Trizol (Sigma-Aldrich), followed by the addition of 1-bromo-3-chloropropane, separation of the aqueous phase, and precipitation of RNA using isopropanol (Sigma-Aldrich) together with GlycoBlue (Thermo Fisher Scientific). The pellet was washed in ethanol (75% in nuclease-free water), air-dried, resuspended in nuclease-free water, and frozen (−80 °C) until later analysis by RT-qPCR (see below).

*Staining procedures*

Sections for histopathological examination were stained using an automated stainer (Leica ST5020; Leica Biosystems, Newcastle, UK). In brief, slides were washed in two changes of xylene (2 min each), washed in absolute ethanol three times (2 min each), rinsed in water, and then stained with haematoxylin (Gill^®^-2; Sigma-Aldrich); then rinsed in water, placed in bluing agent for 2 min, differentiated in acid–ethanol (30 s), and rinsed in water. Slides were then placed in bluing agent (ammoniated water, 1 min), rinsed in water, and stained with ‘yellowish’ Eosin Y (Merck-Millipore, Darmstadt, Germany) for 1 min. Slides were rinsed in water, dehydrated in ethanol, and cleared with xylene before mounting and coverslipping.

For immunostaining for Ki67, sections were washed in three changes of xylene (5 min each), then placed in 100% ethanol and 95% ethanol for 10 min each, followed by deionised water (dH_2_O) for 5 min. Antigen retrieval was undertaken by heating in 10 mm sodium citrate (pH 6.0) for 10 min in a pressure cooker. Slides were cooled for 15 min and then washed three times (5 min each) in dH_2_O. Slides were then incubated in hydrogen peroxide (3% vol/vol; Sigma-Aldrich) and washed twice (5 min each) in dH2O and then wash buffer (Tween in phosphate-buffered saline, 0.01% vol/vol) for 5 min. Slides were blocked with 10% vol/vol goat serum (Abcam, Cambridge, UK) diluted in PBS for 1 h in a humidified chamber at room temperature. The blocking solution was removed and anti-Ki67, rabbit mAb, primary antibody (Catalogue No 12202, Lot 4; Cell Signaling Technology, Danvers, MA, USA), diluted 1:400 (final protein concentration 0.418 µg/ml) in proprietary SignalStain antibody dilution buffer (Catalogue No 8112, Lot 26; Cell Signaling Technology), was added to the slides and left overnight at 4 °C. The next day, slides were washed with wash buffer three times (5 min each), and the primary antibody was detected by incubation at room temperature for 30 min with a horseradish peroxidase-linked goat anti-rabbit antibody prepared in accordance with the manufacturer’s instructions (SignalStain boost detection reagent, Catalogue No 8114, Lot 17; Cell Signaling Technology). Slides were washed in wash buffer three times (5 min each) and then covered in SignalStain DAB chromogen concentrate (#11725, Lot 6; Cell Signaling Technology) diluted in SignalStain DAB diluent (#11724, Lot 6; Cell Signaling Technology) for 20 min. Slides were rinsed in water, counterstained with haematoxylin (Sigma-Aldrich), dehydrated rapidly through graded ethanols, cleared in xylene, and coverslipped. Control slides for Ki67 immunostaining included omission of the primary or secondary antibodies, as well as concentration-matched (0.4175 µg/ml) purified rabbit mAB IgG (Catalogue No 3900, Lot 35; Cell Signaling Technology). All control slides were clear of staining.

To stain with Alcian blue, sections were first dewaxed in two changes of xylene (5 min each). Slides were then placed sequentially in 100% ethanol, 95% ethanol, 70% ethanol solutions (vol/vol in water) and then water for 10 min each. Slides were immersed in Alcian blue solution (1% wt/vol Alcian blue; Sigma-Aldrich) in 3% vol/vol acetic acid in dH_2_O, pH 2.5, for 15 min. Slides were rinsed in running dH_2_O for 5 min, counter-stained with haematoxylin (VWR International, Radnor, PA, USA) diluted 1:8 with dH_2_O, rapidly dehydrated in graded ethanols, and then cleared with two fresh changes of xylene before coverslipping.

In situ *hybridisation (ISH)*

Sections cut at 4 µm thickness were mounted onto Superfrost Plus slides (Thermo Fisher Scientific, Hampton, NH, USA; Catalogue No 12-550-15). Slides were placed into a humidity-controlled 60 °C oven (Thermo Fisher Scientific) for 1 h on the day of the experiment. Custom probes for *Grem1* (Mm-Grem1-O1) generated by Advanced Cell Diagnostics (ACD, Newark, CA, USA) were used in this experiment. This probe consisted of 20 ZZ pairs targeting bases 157–1154 of NCBI Reference Sequence NM_011824.4 for *Mus musculus* *Grem1*. *In situ* hybridisation was carried out using the RNAscope Intro Pack 2.5HD Detection Kit BROWN-Mm (ACD, Catalogue No 322371). The RNAscope Positive Control Probe (Ms PPIB, ACD, Catalogue No 313911) and Negative Control Probe (dapB, ACD, Catalogue No 310043), RNAscope Control slides, mouse 3T3 cell pellets (ACD, Catalogue No 310023), and the HybEZ Hybridization System (ACD) were used according to the manufacturer’s instructions in the kit manuals [Formalin-Fixed Paraffin-Embedded (FFPE) Sample Preparation and Pretreatment for the RNAscope 2.5 Assay, ACD Document No 322452, and RNAscope 2.5 HD Detection Reagent-Brown User Manual, ACD Document No 322310-USM]. The only deviation from the standard protocol was that antigen retrieval was performed as per the alternative method described in ‘Appendix B. Manual Target Retrieval’ of the Formalin-Fixed Paraffin-Embedded (FFPE) Sample Preparation and Pretreatment for the RNAscope 2.5 Assay protocol document (p. 19, ACD Document No 322452).

*Isolation and analysis of mRNA*

Flash-frozen samples of intestine were weighed using a fine balance (Mettler Toledo) and no greater than 30 mg tissue per animal was used for subsequent mRNA extraction. mRNA was extracted using a Qiagen RNeasy Mini Kit (Qiagen, Hilden, Germany), including the on-column DNase digestion (DNase Max Kit, Qiagen). The resulting mRNA was eluted in RNAse-free water and immediately frozen at −80 °C. Total RNA from murine tissues (1 µg) was reverse-transcribed to cDNA using a Superscript III Reverse Transcriptase cDNA synthesis kit (Invitrogen, Thermo Fisher Scientific, Waltham, MA, USA) and real-time qPCR was performed on 384-well plates, with each sample in duplicate. *GusB* (Mm01197698; ABI) was used as an endogenous control according to the Taqman PCR protocol [Applied Biosystems (ABI) Thermo Fisher Scientific, Waltham, MA, USA]. Probes and primers were ordered from ABI as Assay-On-Demand Gene Expression Assays for *Grem1* (Mm00488615; ABI, CA, USA), *Grem2*, and *Nog.* Target mRNA expression was assessed using the standard curve methods and expressed relative to the mean value in the gremlin 1 intact groups.

*Statistical analysis*

Normally distributed data are reported as mean (SD), and the statistical significance of differences between two group means was determined using unpaired *t*-tests. Non-normally distributed data are reported as the median (inter-quartile range), and the statistical significance of differences was determined using the Mann–Whitney rank-sum (unpaired) test. *P* values were computed using the exact (permutation) method. Survival was analysed using Kaplan–Meier estimates of the survival curves and the log-rank test. Proportions were analysed using Fisher’s exact test. *p* < 0.05 was considered statistically significant and where *P* values were greater than 0.001, the exact value is shown. All analyses were undertaken using the Statistical Package for the Social Sciences (SPSS, Version 24; IBM Corporation, Armonk, NY, USA).
