# Supplementary material for: Gremlin 1 depletion in vivo causes severe enteropathy and bone marrow failure
Source: J Pathol. 2020 May 28;251(2):117–22. doi: 10.1002/path.5450 (PMC7384058; doi:10.1002/path.5450)
Supplement: Supplementary file 2 — Supplementary figure legends [file PATH-251-117-s006.docx]

**Gremlin 1 depletion *in vivo* causes severe enteropathy and bone marrow failure**SC Rowan, H Jahns, *et al. J Pathol* DOI: 10.1002/path.5450

**Supplementary figure legends**

**Figure S1. *Grem1* depletion induces widespread changes to the intestinal epithelium and reduces indices of proliferation.** (A–H) Representative images of the jejunum (first and second rows), caecum (third row), and colon (fourth row) of *Grem1^intact^* (first column) and *Grem1^depl^* mice (second column). In the jejunum, the mucosal epithelial lining in *Grem1^depl^* mice consists of disordered, large polygonal cells, often piling up. (B, arrow) These cells had abundant eosinophilic cytoplasm and large oval nuclei with finely stippled chromatin and prominent nucleoli. (B, arrowhead) There was marked anisokaryosis and occasional binucleate or multinucleate cells were seen. (A) In contrast, *Grem1^intact^* mice showed a normal epithelial structure. Panels A, B: H&E staining; scale bar = 25 µm; 40× objective, numerical aperture 0.95. (C, D) Immunostaining for the proliferation marker Ki-67 revealed infrequent, positively stained cells in *Grem1^depl^* mice, whereas in *Grem1^intact^* mice extensive labelling of cells was found in the crypt epithelium. Panels C, D: anti-Ki-67 immunohistochemical staining brown, counterstained with Mayer’s haematoxylin; 20× objective, numerical aperture 0.75. (E–H) Alcian blue staining revealed loss of goblet cells and the presence of some extracellular mucin aggregates in the caecum and colon of *Grem1^depl^* mice when compared with the *Grem1^intact^* mice, where goblet cells were arranged regularly within the crypts. Panels E–H: Alcian blue; scale bar = 50 µm; 20× objective, numerical aperture 0.75.

**Figure S2. *Grem1* mRNA expression in stromal cells throughout the gastrointestinal tract in *Grem1^intact^* mice** (A–F) Representative images of ISH for *Grem1* mRNA in the stomach (A), duodenum (B), jejunum (C), ileum (D), caecum (E), and colon (F) of *Grem1^intact^* mice. Punctate brown labelling for *Grem1* is seen in spindle-shaped cells adjacent to the base of the crypts, along the muscularis mucosa, and in cells within the submucosa and tunica muscularis. Counterstained with Mayer’s haematoxylin; scale bar = 25 µm; 40× objective, numerical aperture 0.75.

**Figure S3.** ***Grem1* mRNA expression was markedly and extensively reduced in the gastrointestinal tract of *Grem1^depl^* mice, although some staining was occasionally evident*.*** Representative images of the intestine of *Grem1^intact^* (first column) and *Grem1^depl^* mice (second column). (A, C) *Grem1* mRNA expression is observed as punctate brown labelling in cells adjacent to the base of the crypts, along the muscularis mucosa, and in cells within the submucosa and the tunica muscularis of *Grem1^intact^* mice. (B, D) Although there was extensive, marked loss of *Grem1* mRNA expression throughout the intestine of *Grem1^depl^* mice (Figure 2, main text), occasional clumping of brown punctate labelling was observed in some cells. ISH for *Grem1* mRNA, counterstained with Mayer’s haematoxylin; scale bar = 20 µm; 60× objective, numerical aperture 0.8.
