# Supplementary figures and images for: Gremlin 1 depletion in vivo causes severe enteropathy and bone marrow failure
Source: J Pathol. 2020 May 28;251(2):117–22. doi: 10.1002/path.5450 (PMC7384058; doi:10.1002/path.5450)

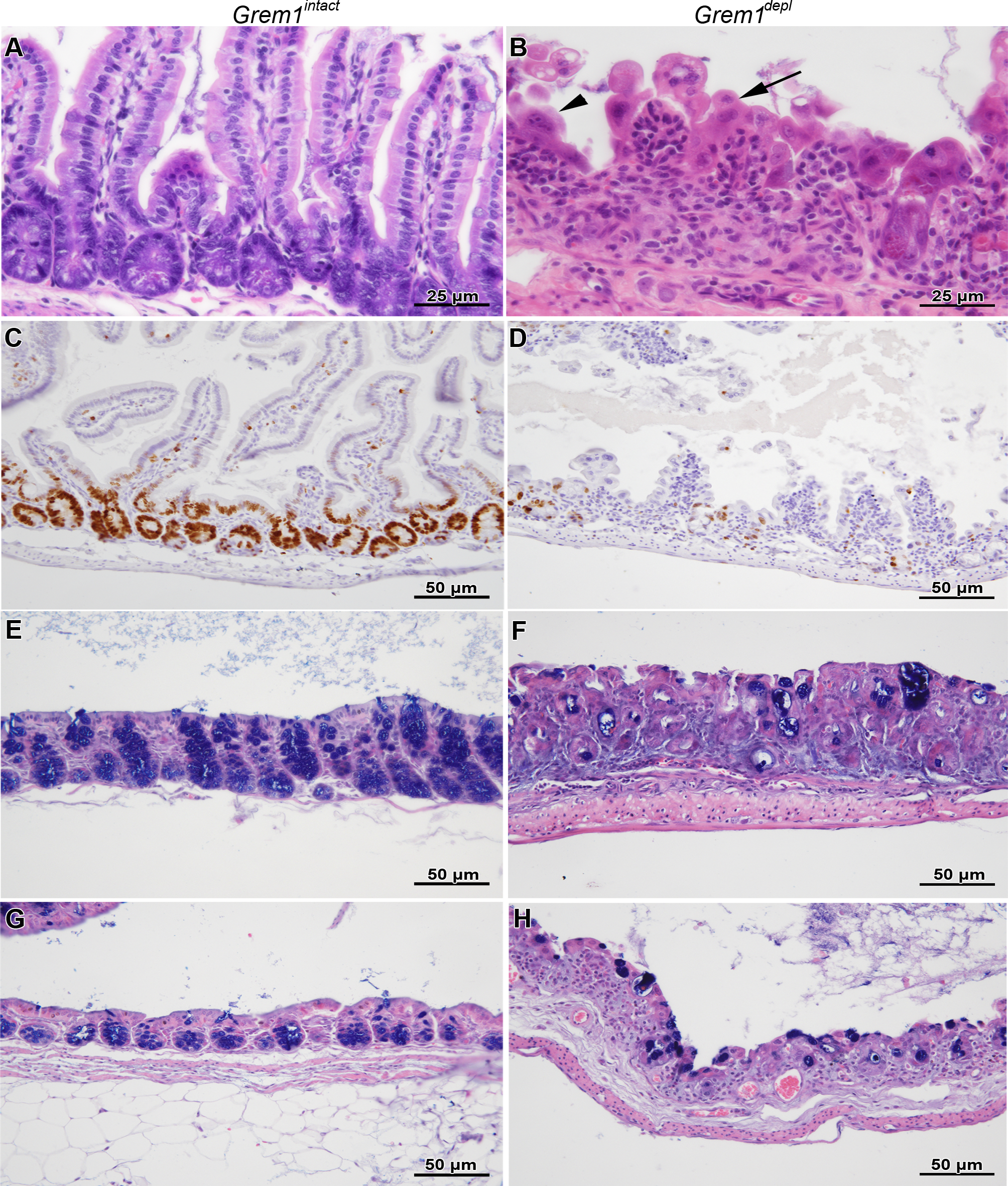

Supplement: Supplementary file 3 — Figure S1. Grem1 depletion induces widespread changes to the intestinal epithelium and reduces indices of proliferation. Representative images of the (A–D) jejunum, (E, F) caecum, and (G, H) colon of Grem1 intact (first column) and Grem1 depl mice (second column) [file PATH-251-117-s003.tif]

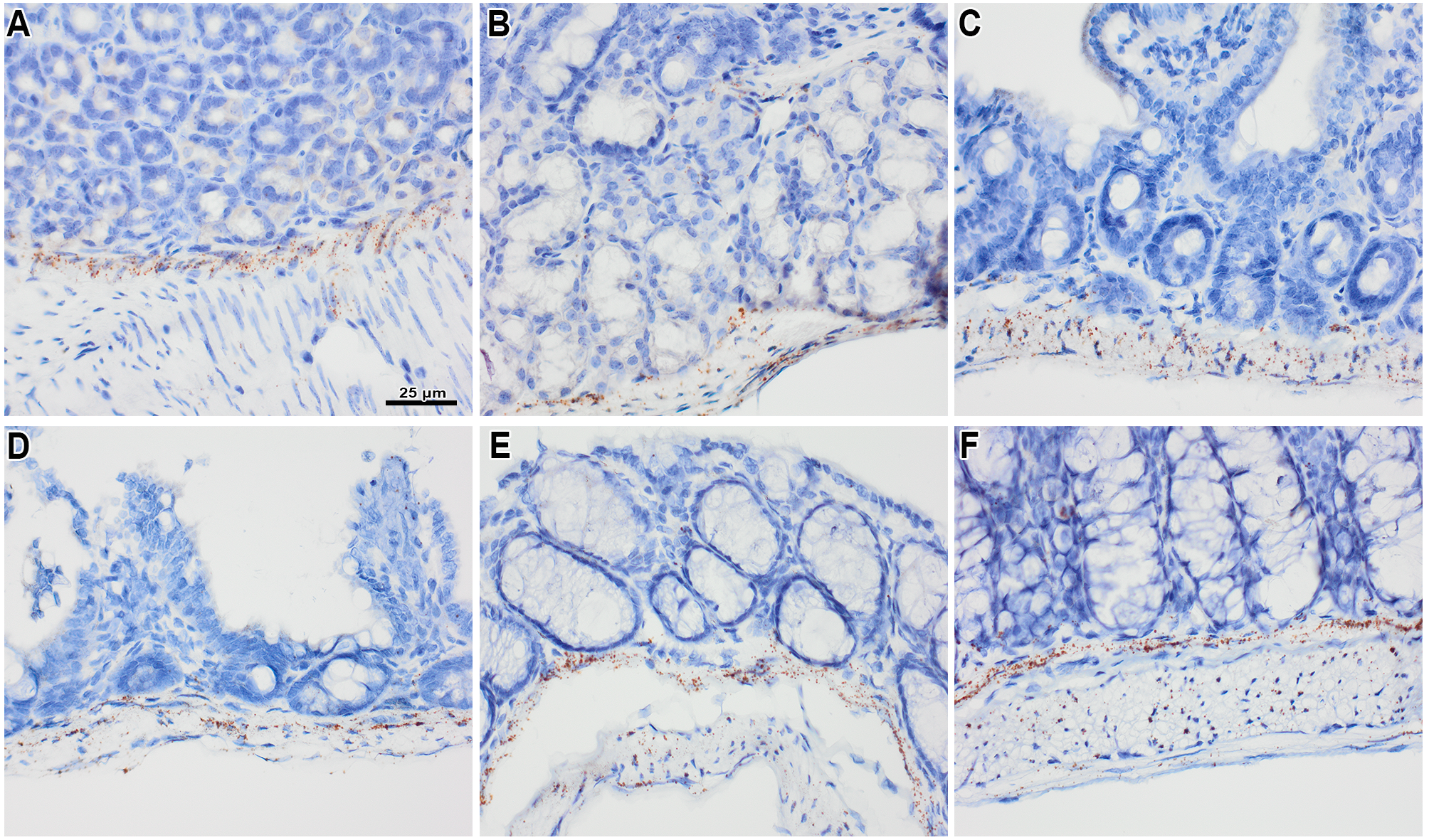

Supplement: Supplementary file 4 — Figure S2. Grem1 mRNA expression in stromal cells throughout the gastrointestinal tract in Grem1 intact mice. Representative images of ISH for Grem1 mRNA in the (A) stomach, (B) duodenum, (C) jejunum, (D) ileum, (E) caecum, and (F) colon of Grem1 intact mice [file PATH-251-117-s004.tif]

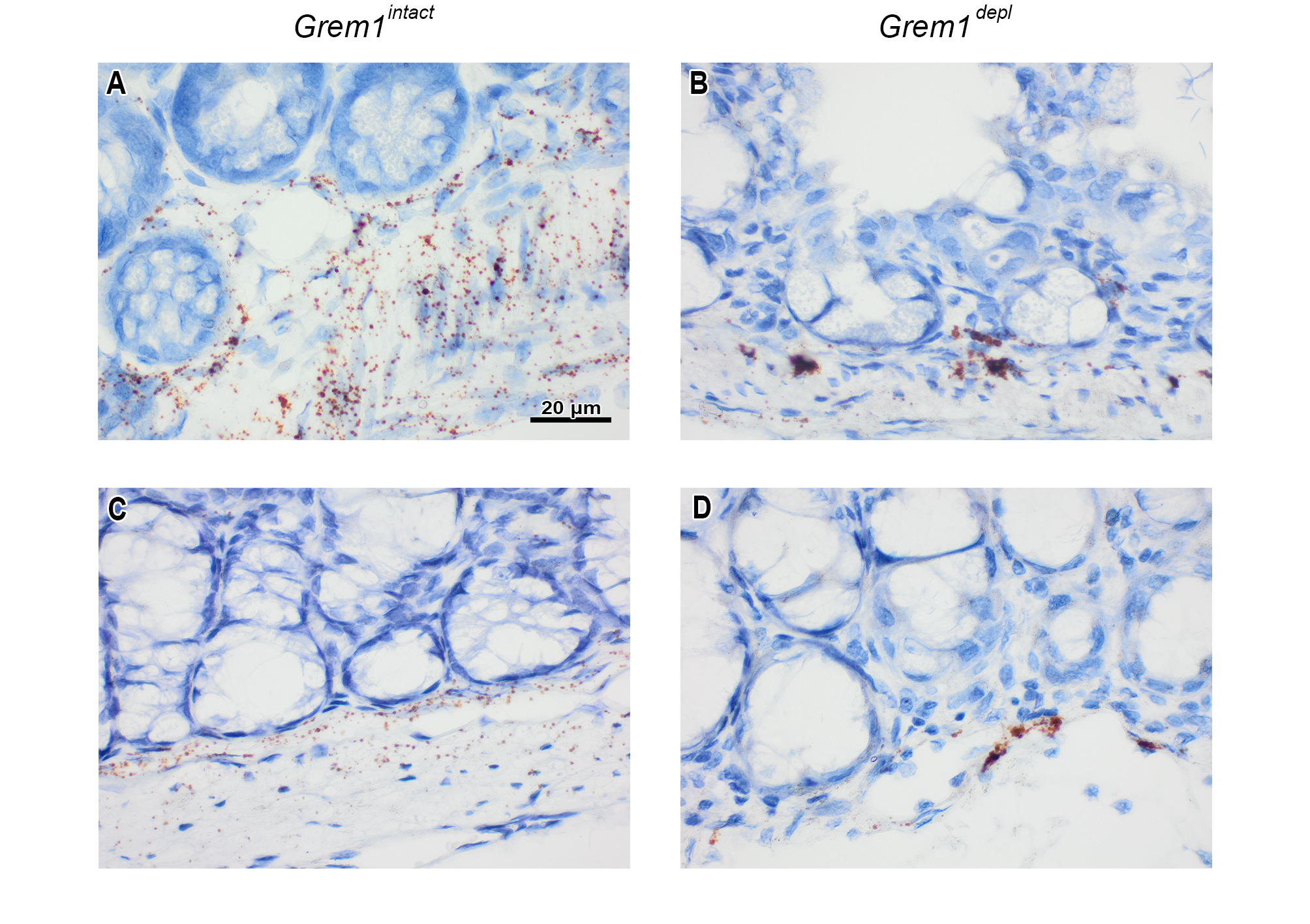

Supplement: Supplementary file 5 — Figure S3. Grem1 mRNA expression was markedly and extensively reduced in the gastrointestinal tract of Grem1 depl mice, although some staining was occasionally evident. Representative images of the intestine of Grem1 intact (first column) and Grem1 depl mice (second column) [file PATH-251-117-s005.tif]
