# Supplementary material for: Gremlin 1 depletion in vivo causes severe enteropathy and bone marrow failure
Source: J Pathol. 2020 May 28;251(2):117–22. doi: 10.1002/path.5450 (PMC7384058; doi:10.1002/path.5450)
Supplement: Supplementary file 6 — Table S1. Results of the serum biochemical analyses Table S2. Results of peripheral blood haematological analyses [file PATH-251-117-s002.docx]

**Gremlin 1 depletion *in vivo* causes severe enteropathy and bone marrow failure**SC Rowan, H Jahns, *et al. J Pathol* DOI: 10.1002/path.5450

**Supplementary tables**

**Table S1.** Results of serum biochemical analyses

|  | ***Grem1^intact^*** | ***Grem1^depl^*** | ***P* value** |
| --- | --- | --- | --- |
| *N* | 6 | 8 |  |
| Total protein (g/l) | 48.8 (1.9) | 48.1 (3.0) | 0.181 |
| Albumin (g/l) | 30.2 (1.5) | 29.3 (1.9) | 0.338 |
| Urea (mm) | 12.0 (1.5) | 8.3 (1.8) | < 0.001* |
| Creatinine (µm) | 38.3 (3.3) | 31.9 (2.8) | 0.002* |
| Glucose (mm) | 13.1 (2.1) | 9.9 (3.2) | 0.054 |
| Cholesterol (mm) | 3.00 (0.4) | 2.0 (0.1) | < 0.001* |
| Alk phos (U/l) | 198.3 (84.0) | 149.1 (40.3) | 0.345 |
| Data are presented as mean (SD). Alk phos, alkaline phosphatase.  *Statistically significant difference (unpaired *t*-test). | | | |

**Table S2.** Results of peripheral blood haematological analyses

|  | ***Grem1^intact^*** | ***Grem1^depl^*** | ***P* value** |
| --- | --- | --- | --- |
| *N* | 6 | 6^†^ |  |
| RBC (× 10^12^ per l) | 8.3 (0.8) | 8.4 (0.6) | 0.890 |
| Reticulocytes (× 10^9^ per l) | 247.8 (27.9) | 68.2 (86.7) | 0.017* |
| Haemoglobin (g/l) | 125.8 (0.3) | 120.3 (12.6) | 0.459 |
| MCV (fl) | 45.5 (1.0) | 42.0 (5.1) | 0.082 |
| MCH (pg) | 15.0 (0.5) | 14.3 (0.6) | 0.162 |
| MCHC (g/l) | 330.0 (4.7) | 280.8 (116.6) | 0.450 |
| Platelets (× 10^9^ per l) | 589.0 (363.4) | 1173.5 (505.8) | 0.052 |
| WBC (× 10^9^ per l) | 4.0 (1.5) | 1.9 (1.3) | 0.037* |
| Neutrophils (× 10^9^ per l) | 0.5 (0.4) | 0.3 (0.3) | 0.463 |
| Lymphocytes (× 10^9^ per l) | 3.4 (1.3) | 1.5 (1.0) | 0.017* |
| Monocytes (× 10^9^ per l) | 0.02 (0.04) | 0.03 (0.04) | 0.242 |
| Eosinophils (× 10^9^ per l) | 0.11 (0.04) | 0.06 (0.07) | 0.262 |

Data are presented as mean (SD). MCH, mean corpuscular haemoglobin; MCHC, mean corpuscular haemoglobin concentration; MCV, mean corpuscular volume; RBC, red blood cell; WBC, white blood cell count.

*Statistically significant difference (unpaired *t*-test).

^†^Two samples lost due to technical difficulties.
